# Comparing the Yield of* Staphylococcus aureus* Recovery with Static versus Agitated Broth Incubation

**DOI:** 10.1155/2018/1462671

**Published:** 2018-07-26

**Authors:** Carol E. Muenks, Patrick G. Hogan, Carey-Ann D. Burnham, Stephanie A. Fritz

**Affiliations:** ^1^Department of Pediatrics, Washington University School of Medicine, St. Louis, MO 63110, USA; ^2^Department of Pathology & Immunology, Washington University School of Medicine, St. Louis, MO 63110, USA

## Abstract

Given the lack of standardization of methodologies for microbial recovery from built environments, we sought to compare the yield of* Staphylococcus aureus* with a broth enrichment method when incubated in agitated versus static conditions. Five unique strains of* S. aureus* at five different concentrations were cultured to compare direct plating, agitated broth enrichment, and static broth enrichment culture methods. All samples were incubated at 35° in ambient air. The lowest concentration recovered across three replicates and five strains did not differ between culture methods (Fisher's exact test, p=0.50); notably, recovery of* S. aureus* was equivalent between static and agitated broth incubation. When broth enrichment was used (both static and agitated), the burden of* S. aureus* growth was higher (by semiquantitative assessment of 4-quadrant streaking) compared to the direct plating culture method. Optimizing strategies for microbial recovery is essential, particularly in areas of lower biomass, given the paucity of research concerning microbial communities of built environments. The results of this study, in conjunction with other experiments investigating microbiomes of built environments, can help inform protocols for standardizing culturing methods within built environments.

## 1. Introduction

There is growing interest in the microbiomes and “resistomes” (the conglomeration of resistance genes) [1] of built environments, such as hospitals, schools, and long-term care facilities [2]. Understanding these microbial communities, their transmission dynamics, and their contribution to person-to-person spread of pathogens could reduce the number of hospital-acquired infections [3]. Standardized methodologies for microbial recovery from the built environment have not been established, making it challenging to compare results between investigations [2]. Current accepted methodologies for cultivating* Staphylococcus aureus* in a laboratory setting recommend broth enrichment but are unclear regarding the necessity of agitated incubation [4, 5]. Incubation with agitation requires specialized equipment, which in high volume studies can be cumbersome. The objective of this study was to determine incubation conditions, specifically whether agitated broth incubation is superior to static broth incubation, yielding the highest recovery of* S. aureus*. 

## 2. Materials and Methods

Using five unique strains of* S. aureus* at five specific concentrations, we compared recovery yield with direct plating versus static broth enrichment versus broth enrichment with agitation. Two of the five* S. aureus* strains were reference strains (ATCC 43300: methicillin-resistant* S. aureus* [MRSA], staphylococcal cassette chromosome* mec *(SCC*mec*) Type II and ATCC 25923: methicillin-susceptible* S. aureus* [MSSA], SCC*mec* negative). The remaining three strains of* S. aureus* were recovered from community-dwelling individuals (abscess drainage specimen: MRSA, SCC*mec* type IV [6]; a colonizing anterior nares isolate: MRSA, SCC*mec* type II [7]; and a colonizing inguinal fold isolate: MSSA, SCC*mec* type IV [7]). Each strain was suspended in normal saline and prepared to a density of 0.5 McFarland Standard. For each strain, five tenfold dilutions were prepared to achieve concentrations ranging from 0 to 10^4^ colony forming units (CFU)/mL. An ESwab (Becton Dickinson, Franklin Lakes, NJ) was submerged in each of the prepared dilutions, then placed into the ESwab transport medium (eluate), and vortexed for 10 seconds. From the eluate, 100 *μ*L of the eluate was then inoculated onto each of three media: trypticase soy agar with 5% sheep blood (blood agar plate [BAP] BBL; Becton Dickinson) and into two tubes of trypticase soy broth (TSB) with 6.5% NaCl (BBL, BD), which were then vortexed for 10 seconds. The BAP and two TSBs (one static, one agitated at 250 rpm) were incubated in an ambient air incubator at 35°C for 24 hours. Following incubation, 100 *μ*L of each TSB suspension was inoculated onto BAPs using the four quadrant streaking method for semiquantitative analysis [8–10] and incubated for an additional 24 hours. Three independent replications of this experiment were performed. Fisher's exact test was performed using IBM SPSS for Windows v23 (Chicago, IL) to compare recovery yield between the three culture methods.

## 3. Results

The range of recovery for each of the three culture methods was the same (10-10^4^ CFU/mL). The lowest concentration recovered across three replicates and five strains did not differ between culture methods (Fisher's exact test, p=0.50); specifically, recovery of* S. aureus* was equivalent between static and agitated broth incubation. When broth enrichment was used (both static and agitated), the burden of* S. aureus* growth was higher (by semiquantitative assessment of 4-quadrant streaking) compared to the direct plating culture method. Both broth enrichment methods recovered* S. aureus* in all four quadrants at 10^4^ CFU/mL, while direct plating only recovered* S. aureus* in the first two quadrants at 10^4^ CFU/mL (**Table 1**).

The range of recovery for both MRSA and MSSA was 10-10^4^ CFU/mL. The lowest concentration recovered for the two reference* S. aureus* strains was 10 CFU/mL, after incubation in static broth. For the two colonizing strains, the lowest concentration recovered was also 10 CFU/mL, occurring six times: once with direct plating, twice with static broth incubation, and three times with agitated broth incubation. The lowest concentration recovered for the infecting isolate was 10^2^ CFU/mL for each of the culture methods.

## 4. Discussion and Conclusions

As interest in the microbial communities of built environments grows, optimal strategies for microbial recovery are essential, particularly in areas of lower biomass. Indeed, the National Academy of Sciences extended a call to close the knowledge gap surrounding how to best investigate the microbiomes of built environments [11]. The present study sought to examine different culturing and incubation methods to best isolate* S. aureus* from built environments. No significant difference in yield of* S. aureus* was observed between static versus agitated broth enrichment, while both broth enrichment methods recovered a higher abundance of* S. aureus *than the direct plating method.

The present findings correlate to previous studies which investigated the role of broth enrichment culture methods in recovering* S. aureus*. Specifically, Mernelius et al. employed both direct plating and agitated broth enrichment incubation culture methods to elucidate the role of broth enrichment in* S. aureus* cultivation within throats of newborn infants and found a 46% increase of yield when broth enrichment was used compared to direct plating [12]. Additionally, Tsang et al. used a static broth enrichment culture method to query the potential underestimation of* S. aureus *carriage recovered with standard direct plating techniques, resulting in a 31% increase of* S. aureus *yield [13].

Although there are limitations to this study including the small sample size and inclusion of only one species (albeit five distinct strains), the insights gathered provide valuable information to the field of study concerning the microbiomes of built environments. This study, in conjunction with the studies outlined above, can contribute to the response to the National Academy of Sciences call [11] to help elucidate protocols for standardized culturing methods within built environments.

## Figures and Tables

**Table 1:** Recovery of *Staphylococcus aureus* by culture method.

*S. aureus* strain	Culture method	Replicate, lowest dilution level of detection			Lowest dilution that was detected for all replicates, CFU/mL
		1	2	3	
Reference ATCC 25923 MSSA, SCC*mec* negative	Direct Plate	10^2^	10^2^	10^2^	10^2^
	Static BE	10	10^2^	10^3^	≤10^3^
	Agitated BE	10	10^2^	10^3^	≤10^3^
Reference ATCC 43300 MRSA, SCC*mec* type IV	Direct Plate	10^2^	10^2^	10^3^	≤10^3^
	Static BE	10^2^	10	10^2^	≤10^2^
	Agitated BE	10^2^	10^2^	10^3^	≤10^3^
Clinical infecting isolate MRSA, SCC*mec* Type IV	Direct Plate	10^2^	10^3^	10^2^	≤10^3^
	Static BE	10^2^	10^3^	10^2^	≤10^3^
	Agitated BE	10^2^	10^2^	10^2^	10^2^
Clinical colonizing isolate MRSA, SCC*mec* Type II	Direct Plate	10^3^	10^2^	10	≤10^3^
	Static BE	10^3^	10^2^	10^2^	≤10^3^
	Agitated BE	10	10^2^	10	≤10^2^
Clinical colonizing isolate MSSA, SCC*mec* Type IV	Direct Plate	10^2^	10^2^	10^2^	10^2^
	Static BE	10	10^3^	10	≤10^3^
	Agitated BE	10	10^2^	10^2^	≤10^2^

CFU, colony forming unit; MRSA, methicillin-resistant *S. aureus*; MSSA, methicillin-susceptible *S. aureus; *SCC*mec, *staphylococcal cassette chromosome *mec; *BE, broth enrichment.

*Note. S. aureus* was recovered at the 10^4^ dilution for all strains and all culture methods at each replicate.

## Data Availability

Access to the data underlying the findings of the study will be considered by the corresponding author upon request.
